# A Collaborative UAV-WSN Network for Monitoring Large Areas

**DOI:** 10.3390/s18124202

**Published:** 2018-11-30

**Authors:** Dan Popescu, Cristian Dragana, Florin Stoican, Loretta Ichim, Grigore Stamatescu

**Affiliations:** Department of Control Engineering and Industrial Informatics, University POLITEHNICA of Bucharest, 060042 București, Romania; cristian.dragana@gmail.com (C.D.); florin.stoican@upb.ro (F.S.); loretta.ichim@upb.ro (L.I.); grigore.stamatescu@upb.ro (G.S.)

**Keywords:** large area monitoring, wireless sensor network, unmanned aerial vehicle, optimal trajectory design, clustering

## Abstract

Large-scale monitoring systems have seen rapid development in recent years. Wireless sensor networks (WSN), composed of thousands of sensing, computing and communication nodes, form the backbone of such systems. Integration with unmanned aerial vehicles (UAVs) leads to increased monitoring area and to better overall performance. This paper presents a hybrid UAV-WSN network which is self-configured to improve the acquisition of environmental data across large areas. A prime objective and novelty of the heterogeneous multi-agent scheme proposed here is the optimal generation of reference trajectories, parameterized after inter- and intra-line distances. The main contribution is the trajectory design, optimized to avoid interdicted regions, to pass near predefined way-points, with guaranteed communication time, and to minimize total path length. Mixed-integer description is employed into the associated constrained optimization problem. The second novelty is the sensor localization and clustering method for optimal ground coverage taking into account the communication information between UAV and a subset of ground sensors (i.e., the cluster heads). Results show improvements in both network and data collection efficiency metrics by implementing the proposed algorithms. These are initially evaluated by means of simulation and then validated on a realistic WSN-UAV test-bed, thus bringing significant practical value.

## 1. Introduction

Wide area monitoring requires a robust and efficient data collection infrastructure, following the latest system design approaches of combining ground sensor nodes and unmanned aerial vehicles (UAVs) [1,2]. Currently, high level research focuses on and emphasizes intelligent data management for various critical infrastructure applications, such as border surveillance or large-scale crop monitoring for precision farming.

In the last years, due to the technological progress and wide spreading of the unmanned aerial vehicles they were introduced into sensor networks thus increasing the performances of large scale monitoring. Many challenges are still open like optimal ground sensor clustering, communication performance, saving energy, and trajectory planning for optimal data acquisition from ground sensors. For example, a cooperative framework for UAVs and ground wireless sensor networks (WSN) taking into account a k-means driven grouping approach, the communication performance, the position, and other factors can lead to higher performances [2]. By enabling the acquisition of site-specific measurements, data processing and local information sharing, WSNs have become one of the most promising technologies for wide area monitoring [3]. However, when WSNs are increasingly being deployed over large geographical areas, the limitations of the individual sensor nodes begin to hinder the overall performance of the monitoring system. It has been established that wireless sensors nodes can easily become overused due to high power consumption when used in processes involving the continuous exchange of data. In addition to energy management and data transfer issues, data bandwidth reliability and time synchronization are also major downsides of WSNs. In terms of energy management, dynamic power and sleep settings are typically required. These are scheduled based on a process runtime timetable, time of day, weather, service schedule, etc. An iterative algorithm based on successive convex optimization technique was recently proposed in [4] for an energy-efficient data collection by means of UAVs. This approach can prolong the network lifetime.

Basically, in order to overcome WSN challenges in large scale monitoring deployments, complementary peripherals can be added. One conventional system architecture uses a fixed sink node surrounded by sensor nodes. This strategy is known as static data collection. Data is uploaded using one-hop or multi-hop routing. High energy consumption within the sensor nodes and shorter life cycle for the sink node must be taken into account.

An alternative strategy involves the use of a ground mobile sink node. The mobile sink node exchanges data with the sensor nodes found on its static or dynamically computed navigation path. This method reduces the energy consumption but shows a significant downside by limiting the mobile collector to flat navigable surfaces. To overcome this limitation, the mobile sink node can be carried by an UAV. Recent research has shown that such a UAV-WSN collaboration enables the widespread deployment of WSNs for large scale monitoring applications.

Of particular importance in optimizing the energy consumption of the UAV network is the trajectory generation. The reference trajectory generation can be partitioned into a couple of distinct stages. First, the entire area of interest is covered in order to retrieve information about the location and strength of the deployed sensors. Second, based on an intermediary step of aggregation (where the sensors are clustered and sensor heads for each cluster are selected), the UAV has to pass near all of the sensor heads in order to retrieve information. In both cases the reference trajectory is based on the notions of flatness and subsequent B-spline parameterization, extensively discussed in [5,6]. The former allows to guarantee the feasibility of the trajectory (in the sense that it respects the dynamics of the UAV) and the latter allows various algebraic and geometric guarantees (e.g., continuity up to a predefined degree, inclusion in to a given convex region).

The UAV network can assume the role of mobile base stations when needed, in order to regroup the sensor nodes placed within the coverage area, into WSN clusters. By means of such subnets measuring the surrounding physical world becomes more focused, more relevant and control oriented over the particular analyzed area, thus ensuring that potentially critical data is not lost. Moreover, smarter measurement systems powered by UAVs embed more processing at the point of acquisition, capturing valuable critical data that in the hands of the right people or proper software solution, can translate to better and faster data-driven decisions.

WSNs that incorporate UAVs can be transformed into applications that offer specific features and benefits such as: (1) On-site data collection and examination: UAVs can be implemented as data agents, capable both to upload data to the network and/or send the data back to base stations [7]; (2) Clustering support: UAVs can support ground WSN clustering mechanism by electing the cluster heads; (3) Sensor data 3D modeling: UAVs can provide mapping information (three-dimensional sampling) about the area of interest. UAVs can be also used to support ground WSN deployment [8]. This brings the flexibility of flying UAVs to any location in the area of interest.

In [9] authors discussed the opportunities of optimized WSN architectures at different design levels, to meet application requirements. The paper is particularly focused on WSN dynamic optimization and proposes a Markov Decision Process (MDP)-based dynamic optimization technique for WSNs to meet application requirements considering the presence of changing environmental stimuli.

More articles on WSN had as main theme the study of long-term monitoring [10,11,12]. The maintenance cost and implementation of the ground WSN are of great importance for long-term environment monitoring. A solution to this problem is proposed in [10] by integrating WSN platform in IoT applications. The same author used a low-cost primary cell of WSN to design and implement a long-term, low maintenance wildfire monitoring WSN platform [12]. Arguably, the main factor in extending network life is energy efficiency [4,13]. A poor management of energy consumption can lead to shorter network life, especially when the sensor nodes are powered by batteries. Depending of the sensor types, a balance between the number of acquisitions and the number of transmitted messages is necessary [13].

To ensure the network efficiency, a UAV trajectory optimization for sensor data gathering is proposed in [4,14]. In case of sparse ground WSN [14] the solution is the minimum path length. In our paper we proposed the minimum path length with obstacle avoidance restriction. The trajectory design in [4] is based on a mixed-integer, non-convex optimization problem. Moreover, the surface features are taken into account for efficient transmission between the sensors and cluster heads at ground level [15,16].

More recently, multiple UAVs-multiple WSNs systems were investigated [17,18]. For data collection with multiple UAVs, the authors in [17] present three trajectory planners: based on genetics algorithm, rapidly-exploring random trees, and optimal rapidly-exploring random trees. A new and promising application of multiple UAVs-multiple WSNs systems is in disaster management and rescue operations [18]. For example, a cooperative heterogeneous framework for UAV-WSN is presented in [2]. The system presented in [19] is composed of a hierarchical, three-layer network architecture: sensor nodes, fixed-group leaders, and an UAV sink node. A ground WSN clustering method based on communication performance, position and other indicators is firstly introduced. Further discussions evaluate the performance of a proposed optimal flight trajectory algorithm from a comparative standpoint, considering other related approaches. Starting from the same idea of heterogeneous layered architecture, and exploring WSN ground clustering methods, the authors in [19] evaluated the performance of two algorithms for optimal selection of cluster heads. Selected cluster heads became waypoints along the UAV trajectory. The Particle Swarm Optimization (PSO) is used to decide the network’s topology in order to reduce the energy consumption, improve communication quality and reduce the travelling time. All the results for proposed algorithm evaluations are obtained through comparative simulations. 

UAV support for heterogeneous sensor network monitoring was also discussed in [20]. The UAV is mainly used as mobile gateway for the ground sensor layer. The main objective was to design effective clustering mechanisms for resource balancing given the constraints introduced by battery operated devices. WSN constraints are thoroughly discussed in [21], with respect to the main optimization problems. 

In [22] the authors analyzed a data collection strategy based on three-layer architecture, using mobile data collectors. The proposed modeling and simulation provide clear guidelines for deployment for sparse WSN. A UAV relay based system for hybrid wireless sensors networks, in the context of emerging 5G telecommunication networks architectures, was presented in [23]. Simulation based modelling validates the optimization approach of UAV path planning with respect to node power and radio channel constraints. 

An event detection framework for cloud WSN with UAV support is discussed in [24]. The proposed algorithm, with cloud backend support, enables on-demand optimal data collection with respect to both flying parameters and ground network deployment. UAV as a mobile node sink within an existing WSN is illustrated in [25] for IPv6 over Low-Power Wireless Personal Area Networks (6lowpan) communication protocols and dynamic clustering. Communication performance of a dual-stack single radio implementation for downstream and upstream links is assessed thus eliminating the need for the UAV to use separate radio interfaces for sensor network and ground control station communication. From an application perspective, the authors in [26] described the implementation of a solar powered UAV in conjunction with a ground WSN for environmental monitoring. Also, UAV-supported WSN have been deployed in coastal areas by placing the sensing nodes on floating buoys [27].

In [28] is approached the opportunity for in-network pre-processing of sensory data and also for the planning of the UAV’s local tasks, aiming at increasing the efficiency of the data collection process. A gradient scheme to support decision-making tasks planning was introduced. Authors proposed and evaluated a general architecture for a wide area monitoring system and the use of decentralized sensor fusion mechanisms for efficient data mining, promising to overcome WSN constraints.

In this paper we proposed a hybrid UAV-WSN system [29] to improve the large area monitoring. We first describe a decentralised multilevel architecture, with an UAV fitted with a sink node, serving as data collector, used to enhance the connection between ground WSNs and central gateway (base station). Considering the random deployment WSN nature and therefore the clustered structure, we focus on describing an effective mechanism for ground WSN discovery, while ensuring certain support for the election of the most appropriate communication links and cluster grouping. Trajectory-wise, we consider two distinct stages. First, we plan a discovery trajectory, based on a photogrammetry algorithm, during which the sensor position is estimated and information about cluster structure is gathered. Second, we plan a trajectory for data acquisition, which passes through the neighbourhoods of the cluster heads, with guaranteed communication time and guaranteed obstacle avoidance.

## 2. Materials and Methods 

To overcome the limits of large-scale WSN monitoring systems and to improve the global system, our solution uses a heterogeneous data collection architecture based on WSN-UAV collaboration. This heterogeneous architecture implies that the UAV will ensure the collection of WSNs data, providing a reliable channel for the transmission of information. Depending on each application and the specific data collection requirements, the UAV can behave as a communication relay to connect WSNs to the remote base station or simply record ground data collected for subsequent analysis.

### 2.1. Monitoring System Architecture

In [29], the advantages of a general architecture for a heterogeneous monitoring system (UAVs-WSNs) are discussed, tailored for large scale monitoring and information processing, through a decentralized fusion approach. Starting from the same decentralized multilevel architecture, a single UAV serves here as data collector for the on-ground sensing nodes clusters and is used to enhance the connection between ground WSNs and the central gateway (base station). Communication-wise, in this paper, we divide this decentralized architecture into several levels, thus, a multilevel architecture. Top-most, a single UAV serves as a data collector for the ground detection nodes which compose the lower levels.

**Figure 1:**
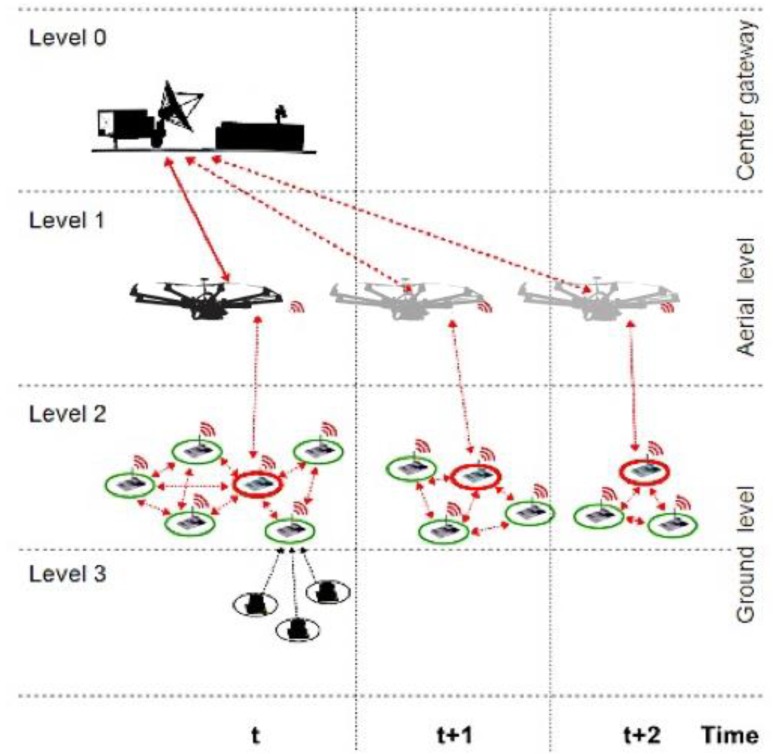
Hybrid UAV-WSN large scale monitoring system concept.

In a multi-level representation, the proposed hybrid UAV-WSN is described as in Figure 1. Starting from the ground level, the monitoring system consists of an extended network of sensors, which can be divided into WSN subsystems, further referred to as clusters. The clustered sensor nodes (Level 2) collect sensory data using specific acquisition equipment (Level 3) and provide efficient data analysis through in-network data processing mechanisms.

The fusion of sensor data is optimally established using effective consensus schemes. Typically, under the consensus mechanism, each sensor node performs gross estimation of the global average value and continues to improve its value through local information exchange between cluster neighbors. The UAV (Level 1) exchanges data with each cluster through a single sensor node further referred as cluster head. Cluster heads are marked with red color in Figure 1. The measure of limiting UAV-WSN data exchange to a single node per cluster is adopted in order to substantially reduce the burden on the communication channel. We assume that the power consumption of intra-cluster communication is much less than the power required for direct sensor-UAV communication.

The relevant sensory information is transmitted by UAV to ground station (Center gateway—Level 0). Additionally, the UAV contributes to the dynamic clustering of ground sensor nodes and cluster head selection mechanism.

Several assumptions about the mission parameters and external environment must be considered:

(1) The nodes are randomly scattered within the area of interest; either due to the lack of GPS coordination or because of perturbing factors such as wind gusts when doing aerial deployment.
(2) The area of interest has restricted regions over which flights are not allowed such as: buildings, roads or other areas where humans are present.

The second item in particular raises some interesting operational limitations for the mission: the interdicted areas are ignored by the sensor network, being transparent from the communication viewpoint, but are seen as obstacles to be avoided from the UAV’s viewpoint.

The overall data gathering problem is divided, due to its complexity and many requirements, into several sequential subproblems:

(1) Assuming that the sensor position is unknown, a preliminary stage in which the UAV(s) gather location data is required. This can be assimilated to a photogrammetry procedure in which the surface of interest is crossed repeatedly to estimate the sensors’ position and receive information about cluster grouping, decided at the WSN level.
(2) Putting together the information about obstacles, cluster heads and communication restrictions, a list of possible reference trajectories for the UAV(s) is obtained.
(3) Lastly, during flight, it is ensured via auto-pilot feedback that the reference trajectories are tracked within prescribed tolerance bounds.

While none of the issues mentioned above is completely new, we consider that the reciprocal influences between each stage are of interest and rise challenging theoretical and practical issues. The paper concentrates on the first two items in this classification. The third is both usually implemented over commonly available “autopilot” software and, in any case, requires access to the inner components of the UAV—which might not be welcomed due to warranty or security reasons.

**Figure 2:**
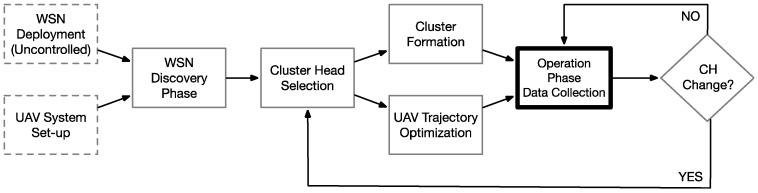
Hybrid UAV-WSN large scale monitoring functional diagram.

Figure 2 summarizes the operational flow of the proposed UAV-WSN large scale monitoring system. In the preliminary phase the WSN is deployed over the target region in an uncontrolled manner and the UAV system is set-up. Subsequently a WSN discovery phase is carried out by the UAV and cluster heads (CH) are selected from the set of ground sensor nodes. The WSN then self-organizes around the selected CHs and UAV trajectories for data collection are optimised. The main operational loop consists of periodically sampling the monitored parameters and relaying the corresponding events towards the UAV. CH changes can occur locally in order to balance the node battery levels or in the case of losing connection to some of the nodes. In this case the CH selection is reinitialized and the process repeats.

### 2.2. Trajectory Planning for the Estimation of Sensor Positions 

As stated earlier, possible methods for the widespread dispersion of sensor nodes involve the use of air vehicles. Such a solution easily leads to an irregular distribution (characterized by a connected graph). Considering this aspect, it is mandatory to define a mechanism to obtain an estimate of the position of each sensor node placed on the ground. We start by defining a flight plan to ensure full coverage of the area of interest. Several specific parameters such as altitude of flying, UAV radio communication range, both for WSN nodes and UAV and others must be considered. 

We consider therefore a multi-obstacle environment a priori known. That is, we consider a collection of obstacles $\;O=\left\{O_{l}\right\}\subset R^{n}$, as illustrated in the proof-of-concept example shown in Figure 2 (while the space dimension is n=3 or even n=2 if we assume the UAVs to fly at constant height, we keep n to highlight that the results hold for a generic space dimension).

**Figure 3:**
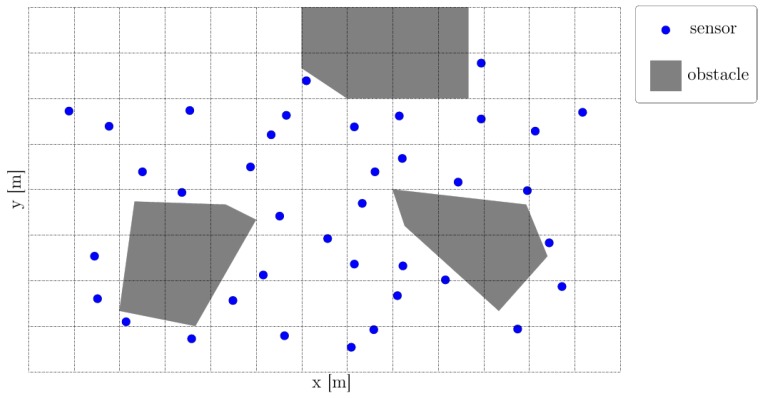
Example of aerial vehicle flight path planning in a multi-obstacles environment.

Within this environment we consider a collection of sensors $S=\left\{S_{j}\right\}$. Each sensor is characterized by its location $z_{j}\notin O$ and communication radius $r_{j}$, the former to be determined at a later step. In Figure 3, a grid of 38 sensors (blue dots) are randomly distributed through the feasible domain (the complement of the union of three restricted areas (gray areas). 

Assuming that the sensors are already deployed we require initial flights which cover the area of interest and, based on the information retrieved from the ground, provide the estimate ${\hat{z}}_{j}$ of the true sensor location $z_{j}$.

For a UAV following trajectory r(t), the estimation of the j-th sensor position is then given as:

(1) $${\hat{z}}_{j}\in {\hat{Z}}_{j}≜\underset{t:S_{j}visible}{⋂}\left\{z:||r(t)-z||\leq r_{j}\right\}.$$

Equation (1) simply states that the sensor’s position has to lie within all the circles centered on the points of the UAV trajectory where signal from the j-th sensor is received.

**Figure 4:**
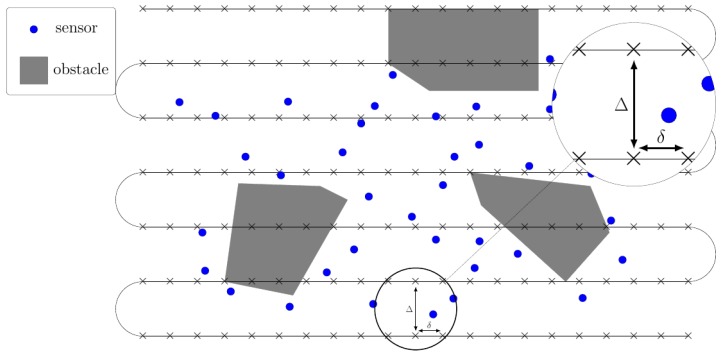
Photogrammetry trajectories for sensor location estimation.

While the UAV trajectory r(t) can be arbitrary, it is much simpler to assume a regular trajectory: we consider a photogrammetry procedure in which the UAV covers the area with parallel lines separated by inter-distance Δ and with measurements done along each line at distance δ as illustrated in Figure 4.

**Figure 5:**
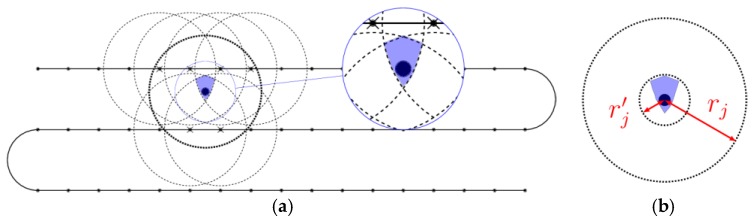
Sensor position estimation through a photogrammetry procedure (with highlight for the uncertainty radius). (**a**) estimation detail; (**b**) uncertainty range.

Applying (1) for one sensor provides its location estimation (the blue region highlighted in the inset), as shown in Figure 5.

Strictly speaking any pair (Δ,δ) which checks $\sqrt{\Delta^{2}+\delta^{2}}<\underset{j}{\min\;}r_{j}$ guarantees that no sensor is missed (it will be “felt” at least once during the flight). In practice, we require that Δ and δ are sufficiently small to obtain a fair estimate of the position. Later on, we require that the UAV spends enough time in the neighborhood of a cluster head. If its position is uncertain we can no longer be sure that communication is ensured. As illustrated in Figure 5b we consider the estimated position of the sensor and find the smallest circle (defined by its center $c_{j}$ and radius ${r_{j}}^{\prime }$) which encloses it. The value of ${r_{j}}^{\prime }$ ultimately depends on the the pair of parameters (Δ,*δ*); thus, these have to be taken of appropriate values in order to obtain an acceptable value for the uncertainty radii ${r_{j}}^{\prime }$.

The pair of center and radius which over-approximated the uncertain region (2) is the result of the optimization problem:

(2) $$\left(c_{j},r_{j}^{\prime }\right)={\mathrm{argmin}}_{c,r}r,\mathrm{such}\;\mathrm{that}\;\hat{Z_{j}}\subseteq C\left(c,r\right),$$

where C(c,r) denotes the circle of center *c* and radius *r*.

### 2.3. WSN Discovery and Clustering Support

Prior to the initial photogrammetry trajectory, the sensor network decides on its cluster partitioning and selects the cluster heads. This information is sent as well to the UAV during its initial flight. Thus, the second purpose of the “flight probe” is to classify and identify the best suitable sensor nodes for the CH position. Sensor nodes are evaluated according to a selection algorithm that uses several relevant indicators:

- the number of unicast messages received by the aerial vehicle from each sensor node *i* denoted *U_i_*, *Ui ∈ M* = {*U*_1_, *…*, *U_n_*}, where *M* is the sensor node set; a set of sensor nodes consists of the individual sensors nodes which reply to the broadcast sequence *t* sent by the UAV towards the ground network;
- the maximum received signal strength indicator (RSSI) value (in dBm) of the unicast messages received from each sensor node *i* denoted RSSI_MAX*_i_,* where *i ∈ M*;
- the minimum link quality indicator (LQI) value of the unicast messages received from each sensor node *i* denoted LQI_MIN*_i_,* where *i ∈ M*;
- the last battery/energy level indicator value (in %) received from each sensor node *i* denoted E*_i,_* where *i ∈ M*;
- the broadcast sequence number *t*, which is used to distinguish nodes belonging to groups selection.

To be able to evaluate the node’s parameters it is necessary to define a dynamic list in the program memory of the UAV’s node. The node structure is composed of unique address, unicast number, signal strength and quality indicator, battery level and broadcast sequence (Algorithm 1).

For ease of implementation and understanding, we have defined a file in which the ground sensor responses are retained, including the unique address, the signal strength (RSSI) at the reception and the time label. 

**Algorithm 1**. Node structure and memory allocation pseudo-code.(1)  **define** the structure of a **node**{ (2)   pointer to the **next_node** (in the list of nodes); (3)   **RIME_address**; (4)   number of unicast messages received **U**; (5)   energy level **last_E**; (6)   maximum RSSI value **RSSI_MAX**; (7)   maximum LQI value **LQI_MIN**; (8)   broadcast sequence **t**; (9)   } (10)  perform **memory allocation** considering the number of (11)  deployed nodes defined node structure; (12)  create the **list of nodes.**

**Figure 6:**
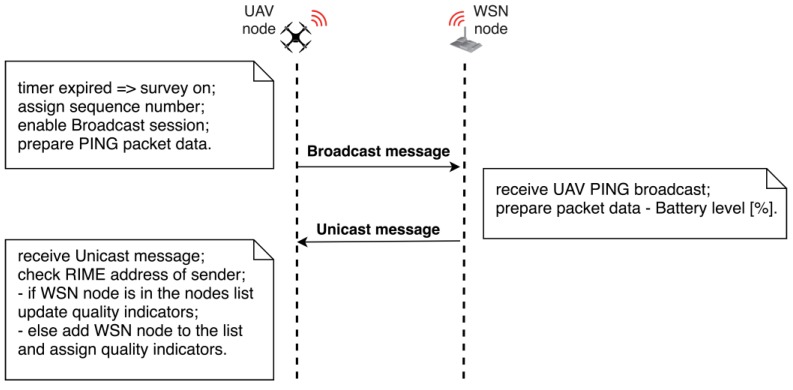
UAV-WSN survey dialog sequence.

The dialogue between sensor nodes is based on the RIME communication stack, using unicast and broadcast messages [30]. Two threads are required for this mechanism. Figure 6 illustrates the sequential diagram of the UAV-WSN survey dialogue. 

**Figure 7:**
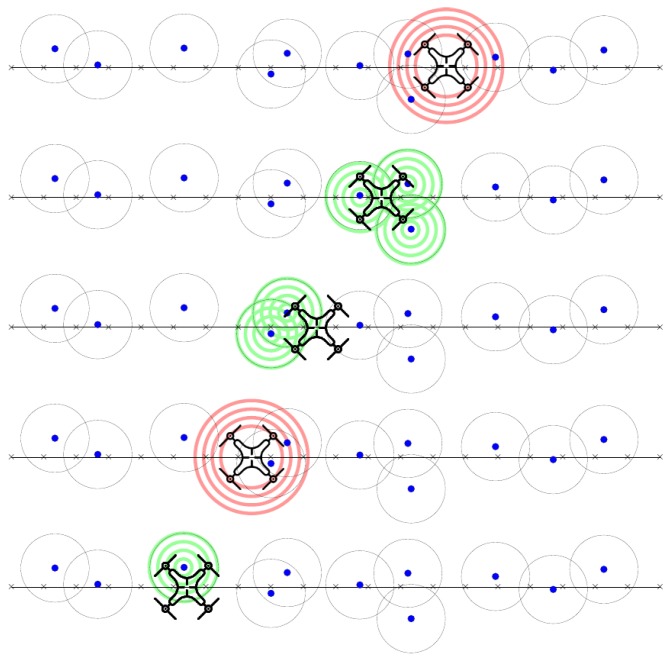
UAV Probe Flight for ground WSN nodes revealing.

The broadcast process (Algorithm 2) manages the broadcast packet transmission sequence. In order to meet the mandatory requirement, to fully cover the area of interest, the broadcast routine is periodically called at a specific sample time, which is closely related to the flight speed. UAV navigation speed during the experiments is around 3 m/s which had been found suitable to allow communication with the ground sensors. Packet collision is handled at the lower stack of the communication protocol using the CSMA/CA mechanism. Given the fact that we use a quadcopter system with no minimum navigation speed this allows good performance for WSN packet collection. Such a broadcast/unicast message exchange sequence is described in Figure 7. Time between broadcast calls is marked with *t*. Unicast responses of sensor nodes are illustrated with green concentric circles and denoted *u_i_*, where *i* is the current number of unicast received from the same ground sensor node at different sequences. Sensor node’s radio cover range is marked with a grey dotted circle. Similarly, UAV’s cover range is marked with red concentric circles.

The UAV enables the broadcast session by time (“survey_ON” is assigned true). A “PING” broadcast message including the broadcast sequence number *t* is sent. The second process thread manages the unicast messages received from ground sensor nodes. When sensor nodes receive a “PING” broadcast message, they respond to the UAV node with a unicast message in which the current battery level is transmitted. Collision avoidance for the unicast message replies is handled natively at the IEEE802.15.4 compliant radio transceiver by means of CSMA/CA protocol (Carrier Sense Multiple Access/Collision Avoidance). In practice this leads to a random communication back-off time for each of the colliding nodes, bounded by the superframe duration of the protocol. As a design choice of the communication architecture, the UAV discovers the ground sensor node positions in the initialization phase Additional data is received by the UAV along with the unicast message that includes the RIME address, the RSSI value, the LQI value.

**Algorithm 2**. Broadcast thread for UAV-WSN discovery (pseudo-code).(1)  **define** the **Broadcast process thread**{ (2)  **begin** the process; (3)  **set up** broadcast connection; (4)  **while** (UAV is in survey_ON) **do** (5)   **Wait (**time**);** (6)    prepare the packet data; (7)   **send broadcast** message; (8)     update last broadcast position; (9)  **while end** (10)   **end** the process; (11) }

We further discuss the routine called when the UAV node receives a unicast message from sensor nodes. First the algorithm (Algorithm 3) decides whether it has already been included in the node list or not. If the RIME address of the sender is not found in the list, a new entry is created and the node structure fields are initialized with the current info. Otherwise, if the node’s address matches a position on the list, the values of interest are updated as follows:

(a) the energy level is updated;
(b) LQI_Min value is updated if the current value is lower than the stored value;

If the stored sequence number *t* is equal to the current broadcast sequence number, the RSSI_MAX value is updated if the current value is greater than the old one and if the *U_i_* parameter is incremented. If *t* is different, then a RSSI comparison is carried out. If the current RSSI value is greater than the stored RSSI_MAX value, the *t* sequence number is updated and the value of the parameter *U_i_* is assigned 1. RSSI is updated as well. On the other hand, if RSSI is lower, only the *U_i_* parameter is updated.

**Algorithm 3**. UAV unicast message received from sensor node function (pseudo-code).(1)  **define** unicast_received(msg, from){ (2)  **for**(every node *i* in list) **do** (3)   **if**(i.addr and from.addr are equal)**then** (4)    update *i* node values: (5)    i.last_E = msg.E; (6)    **if**(current_t = i.t)**then** (7)     **if**(i.RSSI_MAX < msg.RSSI)**then** (8)       i.RSSI_MAX = msg.RSSI; (9)     **end if;** (10)       i.U = U + 1; (11)    **else** (12)     **if**(i.RSSI_MAX < msg.RSSI)**then**
(13)        i.RSSI_MAX = msg.RSSI; (14)        t = sequence_number; (15)     i.U = 1; (16)     **end if** (17)    **if**(i.LQI_MIN>msg.LQI)**then** (18)        i.LQI_MIN = msg.LQI; (19)    **end if** (20)  **end for** (21)  **if**(no address matches from.addr (i==NULL))**then** (22)   add node to the list; (23)   initialise node’s fields; (24)  **end if** (25) }

The captured data analysis starts with dividing the list of sensor nodes based on the ranges appearing in the sequence number *t*. Intervals of such sequences are chosen by taking into account the network size. A cluster head is elected for each group of sensor nodes. The election method consists of a scoring system with different weights for the selection indicators.

This process uses a combined weight *S_i_* for each *U_i_* ∈  *M_tj_* i ∈ {1,…,n}, where *M_tj_* is the set of sensor nodes with the sequence value *t_j_*, where $t_{i}\;\in \;\{t,\ldots ,t+m\}$ and *m* is chosen as according to the size of the network as well. We chose it empirically as to include in the node discovery process a minimum number of ground nodes for each broadcast instance relayed by the UAV. The number of sequences *m* is inverse proportional with the ground sensor network deployment density. *S_i_* is defined as showing in (3):

*S_i_* = ω_1_·*B_i_* + ω_2_·|*RSSI*_*MAX_i_*| + ω_3_·*E_i_* + ω_4_·1/*LQI*_*MIN_i_* (3)

where ω_1_, ω_2_, ω_3_, ω_4_ are the weights for each indicator, and *B_i_* is a binary parameter associated with the sensor *U*_i_ taking the value “1” if the sensor responded to UAV and “0” else. 

Current experiments have used a larger value for ω for the RSSI and energy levels of the nodes but the mechanism is flexible to allow dynamic reconfiguration of the cluster head scoring. The cluster heads are elected for each *M_tj_*, by choosing the sensor node with the maximum *S_i_* value.

### 2.4. WNS Clustering Mechanism

**Figure 8:**
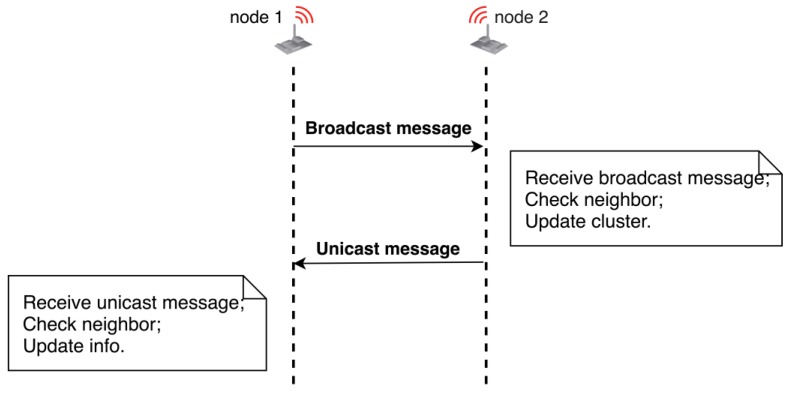
Clustering sequence.

Clustering is a suitable routing technology for large-scale monitoring systems, with a number of advantages such as scalability, data convergence, lower load, low power consumption, robustness [31]. The WSN clustering algorithm described in this paper is conceived from the premise that a number of sensor nodes have been selected and informed by the UAV node about their cluster head role. Thus, Figure 8 illustrates a simplified clustering sequence. The clustering mechanism is based on the RIME communication stack and exchanging of broadcast and unicast messages between neighbour nodes in a random manner. 

Every node randomly broadcasts a message comprising its RIME unique address, current cluster membership, its role in the cluster (CH or simple) and an estimated number of cluster members. This is illustrated in Algorithm 4.

When a neighbor node receives a broadcast message, first it checks whether the sender node is or not in its list of neighbors. If it is not found, then it will be immediately added. Thereafter, it checks the sender information and decides whether to join or not the neighbor group. This decision is made depending on the number of hops to CH, the estimated signal strength (RSSI) and other criteria. This is illustrated in Algorithm 5.

**Algorithm 4**. Broadcast message initiation process for clustering phase (pseudo-code).**Input:** clusterSize, cluster, isCH, hopsToCH
**Output:** Broadcast_msg, seqNo (1)  **enable** Broadcast call back function Broadcast_Recv (2)  **while**(clusterSize < limit) do (3)   **generate** random_time; (4)   **wait**(random_time); (5)   msg.seqNo ← seqNo; (6)   msg.cluster ← cluster; (7)   msg.isCH ← isCH; (8)   msg.hopsToCH ← hopsToCH; (9)   **load** msg; (10)   **send** Broadcast(msg); (11)  **end while**

A node decides to join the sender’s cluster starting from the main following rules: the node is not yet associated to a cluster; the node belongs to a different cluster but the number of hops to the CH is smaller or the number of hops is the same but RSSI is higher.

After decision, the node informs the sender whether it has joined or not the cluster, such that both can update the number of nodes inside the cluster.

**Algorithm 5**. Broadcast message receiving process for clustering phase (pseudo-code).**Input**: msg, cluster, isCH, hopsToCH, clusterSize, CH_RSSI **Output**: Unicast_msg, cluster, isCH, hopsToCH, clusterSize (1)  **If** sender is defined in the neighbor list then     **update** neighbor node data     node.cluster  ← msg.cluster;     node.isCH  ← msg.isCH;     node.hopsToCH  ← msg.hopsToCH;     node.RSSI ← max(RSSI); (2)  **Else** add new neighbor to the list      **initialize** new neighbor data     node.cluster  ← msg.cluster;     node.isCH ← msg.isCH;     node.hopsToCH  ← msg.hopsToCH;     node.RSSI ← msg.RSSI; (3)  **If** cluster = 0 **or** node.cluster ≠ cluster **then** (4)  **If** (node.isCH =1
**and** isCH ≠ 1) **then** (5)    **If** cluster = 0 **or** (cluster ≠ 0 **and** hops = 0 **and** node.RSSI > CH_RSSI) **then**        cluster ← node.cluster;        hops ← 0;        CH_RSSI ← node.RSSI;        clusterSize ← node.clusterSize + 1; (10)   **If** (node.isCH ≠1 **and** isCH ≠ 1) **then** (11)     **If** (hops > 1 **and** hops > node.hops + 1) **or** (hops = 0 and cluster = 0 **and** node.cluster ≠ 0) **then**       cluster  ← node.cluster;       hops ← node.hops + 1;        clusterSize ← clusterSize + 1; **else**
       cluster Size $\leftarrow \sum_{i=1}^{N_{j}}clusterSize/N_{j}$, where $N_{j}$ is the number of neighbors which belong to the same cluster as the sensor node. (12)   msg.clusterSize  ← clusterSize;    msg.cluster  ← cluster; **load** msg; **send** Unicast(msg);

After receiving a unicast message, the node checks if the sender is included in the neighbor list and updates or adds the sender’s information. The next step is to update the cluster size information. This routine is described in Algorithm 6. Note that cluster size information is only roughly calculated using an average consensus estimation. Average consensus estimation refers to the fact that each node within a cluster updates its local value for cluster size based on the values reported by its known neighbors. This occurs without the existence of a central entity propagating this value throughout the network but by means of a distributed process of message exchange among neighbors. Cluster sizes might vary during the deployment of the network as nodes might become unavailable or the UAV might reinitiate the CH assignment process.

**Algorithm 6**. Handling of unicast messages by the UAV function (pseudo-code).**Input**: msg, clusterSize, cluster **Output**: clusterSize (1)  **If** sender is defined in the neighbor list then **update** neighbor node data     node.cluster ← msg.cluster;    node.clusterSize ← msg.clusterSize; (2)  **Else** add new neighbor to the list     **initialize** new neighbor data     node.cluster ← msg.cluster;     node.clusterSize ← msg.clusterSize; (3)    clusterSize $\leftarrow \sum_{i=0}^{N_{j}}clusterSize/N_{j}$, where $N_{j}$ is the number of neighbors which belong to the same cluster as the sensor node.

### 2.5. Trajectory Design for Data Gathering

For further use we recapitulate some of the basic notions of B-spline parameterizations [32]. The B-spline of order d is described by the following recurrence relations (4) and (5):

(4) $$B_{i,1}\left(t\right)=\begin{array}{l} \{\begin{array}{c} 1,for\;\tau_{i}\leq t<\tau_{i+1}\\ 0\;\mathrm{otherwise} \end{array} \end{array},$$

(5) $$B_{i,d}\left(t\right)=\frac{t-\tau_{i}}{\tau_{i+d-1}-\tau_{i}}B_{i,d-1}\left(t\right)+\frac{\tau_{i+d}-t}{\tau_{i+d}-\tau_{i+1}}B_{i+1,d-1}\left(t\right)$$

for d>1,  i=0,1…n=m−d and the non-decreasing time instants (6)

(6) $$T=\left\{\tau_{0}\leq \tau_{1}\leq \ldots \leq \tau_{m}\right\}$$

forming the so-called *knot-vector*.

Taking a collection of *control points* (7)

(7) $$\mathbb{P}=\left\{p_{0},p_{1}\ldots p_{n}\right\}$$

we describe a *B-spline curve* as a linear combination of the control points (7) and the B-spline functions (4), (5) as (8):

(8) $$z(t)=\sum_{i=0}^{n}B_{i,d}(t)P_{i}={PB}_{d}(t)$$

with $P=\left[\begin{array}{l} p_{0}\ldots p_{n} \end{array}\right]$ and $B_{d}(t)={\left[B_{0,d}\ldots B_{n,d}(t)\right]}^{T}$.

Using (8) we may then express the inputs and states of the UAV dynamics in terms of the flat output z(t) and its derivatives (9):

(9) $$x\left(t\right)=\Theta \left(z\left(t\right),\dot{z}\left(t\right),\cdots ,z^{\left(q\right)}\left(t\right)\right)=\;\tilde{\Theta }\left(P,B_{d}\left(t\right)\right),\;u\left(t\right)=\Phi \left(z\left(t\right),\dot{z}\left(t\right),\cdots ,z^{\left(q+1\right)}\left(t\right)\right)=\;\tilde{\Phi }\left(P,B_{d}\left(t\right)\right)$$

where q is the maximum order of derivatives for z(t) and where mappings $\;\tilde{\Theta }\left(\cdot ,\cdot \right),\;\tilde{\Phi }\left(\cdot ,\cdot \right)$ express the states and inputs in terms of the control points P and B-splines $B_{d}\left(t\right)$.

The main difficulty of the mission is to efficiently cover the sensor heads such that all the information they provide is received. Note that this requirement imposes not only to pass near the node heads but also to stay enough time inside their communication range (such that the data is received).

The classic B-spline parameterization allows to pass though (or within a pre-defined neighborhood) of a way-point (the position of the cluster head of interest). The two principal issues are that both the total time for the trajectory and the intermediate times at which the sensor is passing near the way-points have to be a priori given [33]. In here we concentrate on the second issue but mention some heuristics for the first one as well.

First, we consider an iterative scheme where, for fixed way-points and a given permissible velocity interval, we estimate a total time for the UAV trajectory. Second, building on some preliminary work done in [33], we ensure that the UAV spends sufficient time inside the communication radius of each sensor head.

Typically, a collection of *N* + 1 way-points with associated time stamps is considered (10):

(10) $$W=\left\{w_{k}\right\}\;and\;T_{W}=\left\{t_{k}\right\}.$$

Then, the UAV must pass through each way-point *w_k_* at the pre-specified $t_{k}$, that is, $y\left(t_{k}\right)=w_{k}$ (where y(t)  is the position component of the UAV state). These way-point constraints are needlessly restrictive and consequently impact negatively the trajectory generation procedure (even making the entire procedure unfeasible).

Previous results [33] partially alleviate these limitations by first relaxing the spatial restriction (the UAV position has to stay within a neighborhood of the way-point) and secondly by relaxing the temporal restriction (the time at which the UAV passes through the region of interest is not fixed a priori).

Let us associate to each way-point a communication region $S_{k}\subset {\mathbb{R}}^{n_{x}}$ (we consider this region to be a polyhedral set). Then, inclusion (11):

(11) $$\;\tilde{\Theta }\left(B_{d}\left(t\right),P\right)\in \left\{w_{k}\right\}⊕S_{k},\;\forall t\in \left[t_{k}^{-},t_{k}^{+}\right],\forall k=0\ldots N,$$

denotes the fact the trajectory spends enough time (in the interval $\left[t_{k}^{-},t_{k}^{+}\right]$, such that $t_{k}^{+}-t_{k}^{-}\geq t_{min}$ with $t_{min}$ an a priori fixed minimal time interval) near each of its cluster heads (characterized by position $w_{k}$ and communication region $S_{k}$). 

Condition (11) is impractical since it requires the trajectory validation along a continuous time interval (i.e., $t\in \left[t_{k}^{-},t_{k}^{+}\right]$). To alleviate this issue we consider a sampling of the time interval by choosing $\left\{t_{0}\leq {\tilde{t}}_{0},\ldots {\tilde{t}}_{\tilde{N}}\right\}\subset \left[t_{0},t_{N}\right]$.

**Remark** **1.** *Choosing the sampling time interval (${\tilde{t}}_{k+1}-{\tilde{t}}_{k}$) is an important prerequisite: taking the step too large makes the problem formulation too rigid and taking it too small needlessly increases the computational complexity. For an appropriate choice, both the dimension of the sensing regions $S_{k}$ and the bounds on the UAV’s velocity need to be taken into account*.

The inclusion constraint (8) is discretized along the sampling instants and becomes:

(12) $$\;\tilde{\Theta }\left(B_{d}\left({\tilde{t}}_{j}\right),P\right)\in \left\{w_{k}\right\}⊕S_{k}⊕\;\left\{M{\tilde{\alpha }}_{k,j}\right\},\forall k=0\ldots N,$$

where ${\tilde{\alpha }}_{k,j}\in \left\{0,1\right\}$ are binary variables associated to the k-th sensing region $S_{k}$ and the j-th sampling time ${\tilde{t}}_{j}$ and M is a diagonal matrix of appropriate size whose diagonal entries are sufficiently large. Variables ${\tilde{\alpha }}_{k,j}$ can be either “0” or “1”: whenever it is “0” we have that the constraint is verified and whenever is ‘1’ the constraint is ignored (due to the presence of the “big-M” term).

**Remark** **2.** *Note that we do not specify when should the constraint be validated (we do not fix $t_{k}^{-}$ and $t_{k}^{+}$). (11) holds (or not), depending on the value taken by the associated binary variable ${\tilde{\alpha }}_{k,j}$*.

**Proposition** **1** [5]. *For the given way-point and time-stamps collections (7), condition (8) is validated if:*

(13) $$\sum_{j=0}^{\tilde{N}}(1-{\tilde{\alpha }}_{k,j})\cdot ({\tilde{t}}_{j+1}-{\tilde{t}}_{j})>t_{\min},\forall k=0\ldots N,$$

*where* ${\tilde{t}}_{j}$ *and* ${\tilde{\alpha }}_{k,j}$ *are taken as in (12)*.

Proposition 1 does not ensure that the time instants selected through the binary variables $\alpha_{k,j}$ are contiguous. To solve this issue, we impose additional constraints on the binary variables.

**Corollary** **1.** *To ensure that a contiguous time interval of τ time instants is selected by (13) we consider the following inequalities (14)–(17):*

(14) $$\alpha_{k,j}=\beta_{k,j}-\gamma_{k,j},$$

(15) $$\beta_{k,1}\leq \ldots \leq \beta_{k,\tilde{N}},$$

(16) $$\gamma_{k,1}\leq \ldots \leq \gamma_{k,\tilde{N}},$$

(17) $$\gamma_{k,j-\tau }\leq \beta_{k,j},$$

*for all*k=0,…,N*and for all*$j=0,\ldots ,\tilde{N}$.

**Proof.**  (15) and (16) ensure that the binary variables are ordered along the time samples. That is, for each sequence ($\left\{\beta_{k,1}\ldots \beta_{k,\tilde{N}}\right\}$ and $\left\{\gamma_{k,1}\ldots \gamma_{k,\tilde{N}}\right\}$), once there is a switch from “0” to “1”, the value can no longer be changed. (17) ensures that the switch from “0” to “1” happens at latter time for *γ*: if the sequence of variables *β* becomes “1” at index *k* then *γ* can become “1” no earlier than index *k*+*τ*. Lastly, considering (13) it follows that sequence $\left\{\alpha_{k,1}\ldots \alpha_{k,\tilde{N}}\right\}$ will always define a contiguous sequence of values “1” of length at least *τ*. □

Adding the constraints of Corollary 1 to Proposition 1 means that we impose that once the trajectory enters the *k*-th sensing region it will not depart it for at least *τ* consecutive instants of time. Thus, it is in fact possible to replace (13) with (18):

(18) $$\sum_{j=0}^{\tilde{N}}(1-{\tilde{\alpha }}_{k,j})>1,\forall k=0\ldots N$$

A couple of remarks are in order.

**Remark** **3.** *Note that τ consecutive binary variables means that the UAV lies in the k-th sensing region for the time interval:*${\tilde{t}}_{j}\ldots {\tilde{t}}_{j+\tau }$. *In other words, τ should be chosen such that the minimal communication time t_min_ is verified, e.g., assuming that the sampling time T is constant (*$T={\tilde{t}}_{j+1}-{\tilde{t}}_{j},\forall j$*)*, we have**$\tau =\left\lfloor \frac{t_{min}}{T}\right\rfloor$.

**Remark** **4.** *Another aspect of interest (not followed here) is to maintain a constant (or at least bounded) velocity for the UAV. One possible solution is to use Corollary 1 from [21] to express the velocity bounds as a mixed-integer problem (the bound validation has a geometrical interpretation: the velocity vector has to lies in between two concentric circles).*

## 3. Results

### 3.1. Simulations

In what follows we consider the previously introduced environment with multiple obstacles and randomly positioned sensors. We apply the earlier theoretical results to give both location estimations and generate a trajectory which passes efficiently (do to its minimum length) near the cluster heads and, in the same time, avoids the obstacles.

#### 3.1.1. Sensor Location Estimation and Trajectory Planning

To highlight the procedure for sensors’ location estimation, in Figure 8 we illustrate the estimation results for a choice of parameters Δ=100 m,δ=50 m and $r_{j}=150\;m,\forall j$. Recalling the construction of the uncertain region from Section 2.2 we proceed as follows: at each point (denoted by red “x” symbols in Figure 8) along the trajectory where measurements are taken, the UAV gathers information from the sensors and, using its current position and the communication radius $r_{j}=150\;m$ provides an uncertain region ${\hat{Z}}_{j}$ for each of the sensors as in relation (1).

**Figure 9:**
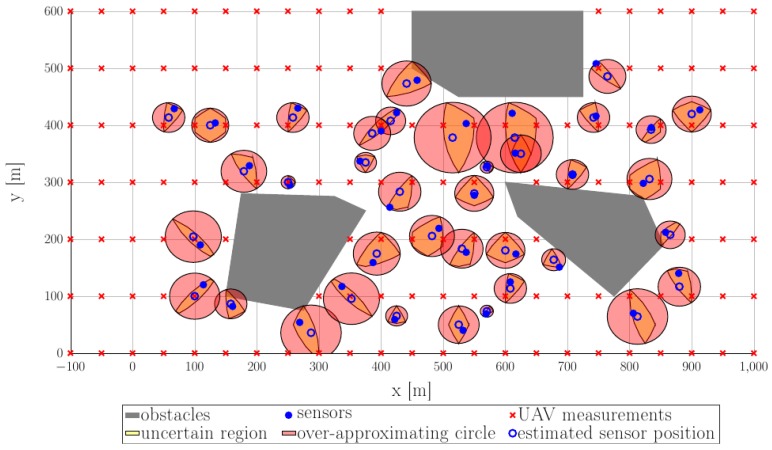
Estimation of the sensor location in both exact and over-approximated (by circles).

As can be seen in Figure 9, the real sensor position (denoted by blue-filled circles), lies inside the yellow-filled uncertain regions (1). These, in turn, are over-approximated, as shown in optimization procedure (2), through circles (the red-filled shapes) characterized by centers $c_{j}$ (hollow blue circles) and radii ${r_{j}}^{\prime }$ as illustrated earlier in Figure 5.

**Remark** **5.** *Note that the assumption of a known communication radius leads to a bounded uncertainty: while the actual position of the sensor is unknown it is, nonetheless, guaranteed to lie within the bounds of uncertain region (1).*

For this particular example we observe that the uncertainty radii ${r_{j}}^{\prime }$ stay in the interval [10.51 m, 61.97 m]. Since the worst estimation, $\;r_{j}^{\prime }=61.97\;m$ is significantly less than the communication radius $r_{j}=150\;m$ we consider the identification procedure to be precise enough. Note as well that the worse uncertainties are for sensors near the obstacles. This happens because we assumed that no measurements are taken above the interdicted regions which means that there are fewer possibilities to assess the sensors’ position. 

Note for further use that the guaranteed communication region is given by the circle centered in $c_{j}$ with radius ${\hat{r}}_{j}=r_{j}-{r^{\prime }}_{j}$. This allows later to consider the sensors as if they are centered in $c_{j}$ with radius ${\hat{r}}_{j}$ from the point of view of the subsequent trajectory generation procedures. The “worst” or “best” estimates are obtained a posteriori: once the UAV has done its initial passing, we proceed to bound the actual sensors’ position through their associated uncertain regions (1) and to subsequently over-approximate these by circles of radii ${r_{j}}^{\prime }$. If the approximation is unsatisfactorily (e.g., ${r_{j}}^{\prime }$ is comparable in size with the communication radius $\;r_{j}$) we need to repeat the procedure with modified parameters (smaller inter-line Δ, and intra-line spacings δ).

**Figure 10:**
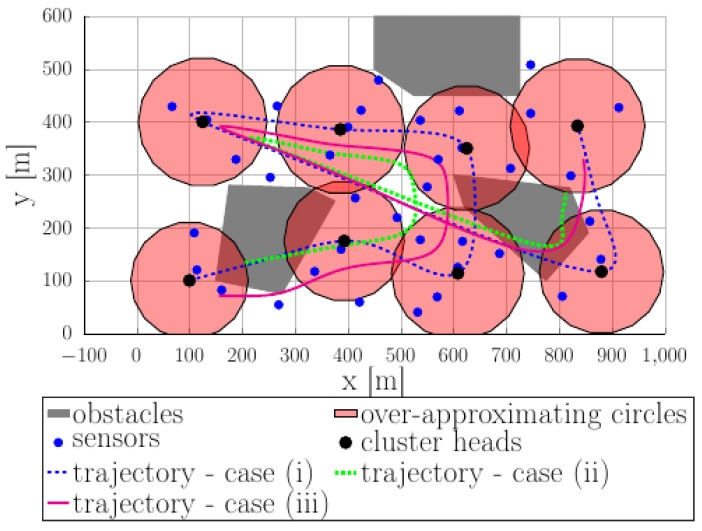
Computed trajectories passing through or near the cluster heads: gray-filled regions—obstacles, semi-transparent red regions—communication areas.

Figure 10 shows various trajectories passing through or near the cluster heads. These trajectories are obtained as per the procedure highlighted in Section 2.4. Briefly: we assume that each trajectory is a weighted sum of basis functions (in our case, the B-splines defined in (4)–(6)) whose weights are the result of a constrained optimization procedure (the cost is the total trajectory length and the constraints are taken from (7)–(18)). We enumerate them in increasing order of complexity:(i) the dashed blue curve describes a trajectory which passes through the cluster heads (large black dots) ignoring the obstacles; (ii) the dotted green curve shows the slightly more complex case where the trajectory is required to pass through the neighbourhood of the cluster head (i.e., to be inside the communication radius—semi-transparent red regions), still ignoring the obstacles; (iii) the magenta solid curve shows a trajectory which ensures both the sufficient communication time constraint and the obstacle avoidance constraint. 

For both cases, in the background are the obstacles (gray-filled regions), the sensors (blue dots) and the cluster heads (denoted by their estimated position—large black dots and estimated radius of communications—semi-transparent red regions).

#### 3.1.2. Clustering

Experiments are based on TelosB/Tmote Sky platforms compatible with Contiki OS. We took advantage of the new available virtual tools for WSN prototyping, the simulation environment Contiki COOJA. The clustering results are obtained by implementation of the deterministic clustering mechanism presented in Section 2.4, with CH assignment by the UAV system. 

**Figure 11:**
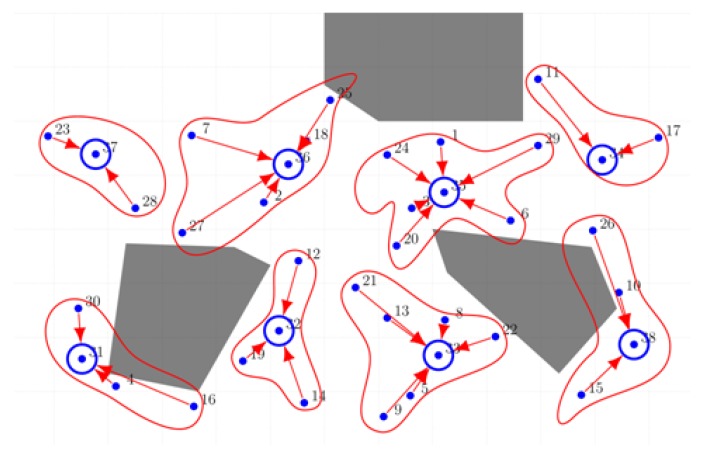
Clustered WSN—Simulation result (normal and head sensors with cluster highlighting).

Regarding the selected CHs, we imagined a navigation path over the nodes diagram and we arbitrary chosen the CHs as they would have been elected with the UAV’s support using the scoring mechanism. Figure 11 illustrates a clustered WSN comprising the same deployment used as the one used for estimating the sensors’ location and computing the trajectory planning. The layout of the network was obtained based on the simulation results of the proposed clustering mechanism. The network has divided in eight clusters with sizes ranging from three to seven sensory nodes. Cluster heads are circled with blue lines.

In addition to the measured time required for WSN clustering, we made an online estimate of the power consumption for each sensor node using the powertrace feature available for Contiki OS, which let us know the time spent in the following states: CPU (active), LPM (low power mode), Transmit and Listen. Some results of simulations made on networks of different sizes are shown in Table 1. The standard deviation value reflects a large variation in both energy consumption and time. This is a predictable result due to the random mechanism and the uneven distributed topology. Mean power increases with WSN’s expansion, while time required for the clustering mechanism is not affected, in fact clustering speed is increased because network density was increased and a large number of messages were exchanged at the same time.

### 3.2. Method Validation on Real Test-Bed 

In order to further evaluate the proposed solution for UAV and ground WSN interlinking, some real implementations were developed. Lately we have paid a particular attention to this topic, involving significant efforts to create a real test-bed for validating the proposed strategies. This section showcases some of our experimental results drawn from test-beds simulations. Our test-bed experiments are based on TelosB/Tmote Sky platforms compatible with Contiki OS. 

Regarding WSN discovery mechanism for estimating positions of sensor nodes, we created a test-bed comprising of five sensor nodes, CM3300, which is an IEEE 802.15.4 compliant wireless sensor node, designed by Advanticsys, based on the original open-source TelosB platform. The main specifications of the CM3300 mote are available in Table 2. 

**Figure 12:**
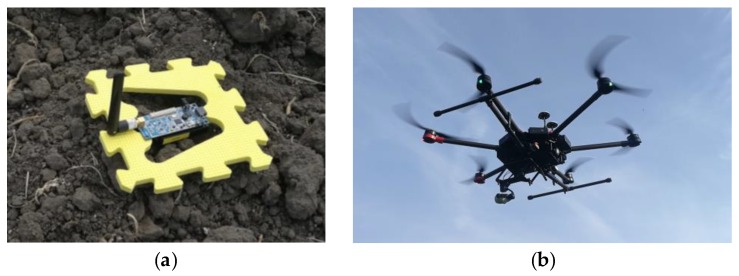
Equipment used in the experimental run. (**a**) Ground sensor node; (**b**) UAV.

Outdoor communication range values are specified in ideal conditions using a power amplifier and a high gain omnidirectional antenna. In a real environmental monitoring application, due to obstructions and vegetations, the expectation is for this to decrease by a factor between 4× and 10×. Four sensor nodes were configured to act as ground sensor nodes according to the described algorithms. To make it easier to spot them, they were placed over yellow colored boards (Figure 12a). The fifth one was configured to act as the UAV node and it was included in the UAV’s payload (Figure 12b). Specifications regarding the UAV used—DJI MATRICE 600 PRO—are available in Table 3.

**Figure 13:**
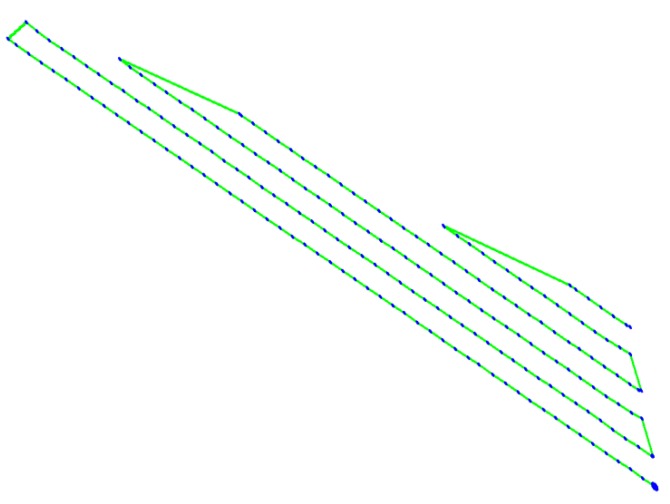
Flying path for the experimental run.

For this experiment, a flight plan was defined, with an elevation of 35 m above the ground, designed to explore an area of interest of 3 ha. The average cruise speed was 3 m/s, and the flight time was 10:47 min. Using the available flight record we generated the following flying path illustrated in Figure 13.

**Figure 14:**
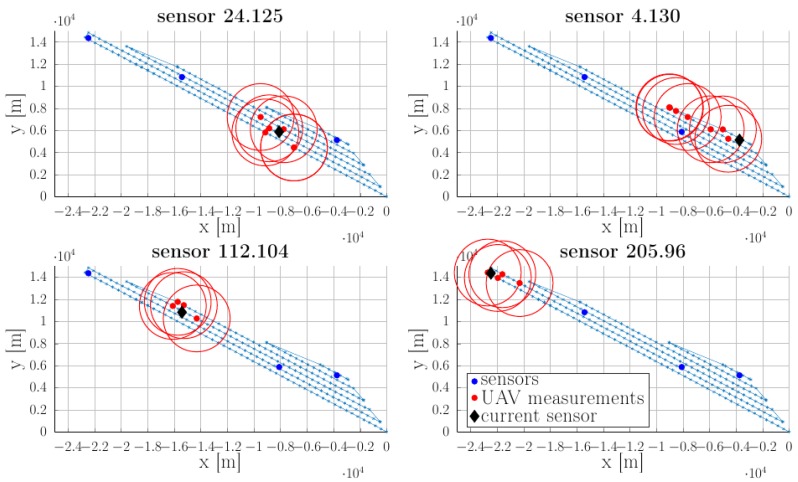
Sensor nodes response positions.

After processing the logged data from the UAV’s node, the following representations for WSN’s response positions could be generated (Figure 14). In each representation, the currently referred sensory node is marked with a large black dot. The red dots represent UAV positions at which packets from the current sensor are received.

To simplify the representation, only responses with a signal strength indicator above a sensitivity limit were retained. Sensor nodes are uniquely identified inside the network by means of their RIME communication stack addresses. For this particular example we observe that better placed nodes inside the area described by the flying path (24.125 and 112.104) can be accurately estimated, while nodes located at the border get the worst estimates. This was previously highlighted in Section 3.1.1.

**Figure 15:**
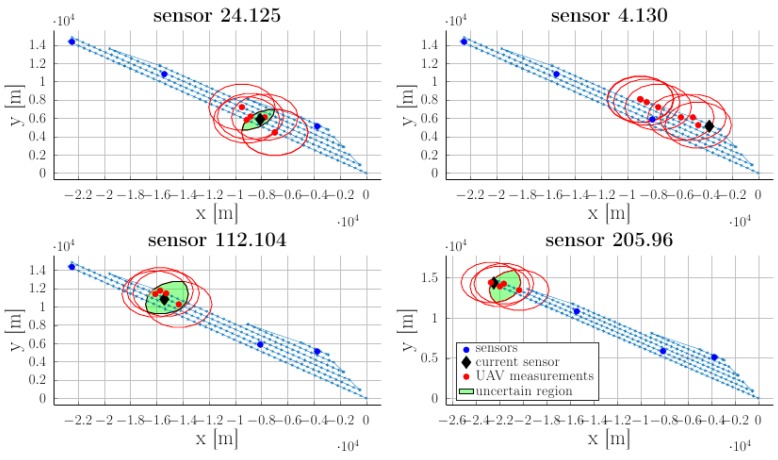
Sensors’ estimated location as the result of succesive UAV mesurements.

Sensors’ estimated location over-approximated by ellipsoidal shapes are illustrated in Figure 15. For node 4.130 which is located at the edge of the monitored area, it was not possible to define an estimated position. For the rest of the nodes we consider the identification procedure to be precise enough (the uncertain radius $r_{j}^{\prime }$ is significantly smaller than the communication radius $r_{j}$).

**Figure 16:**
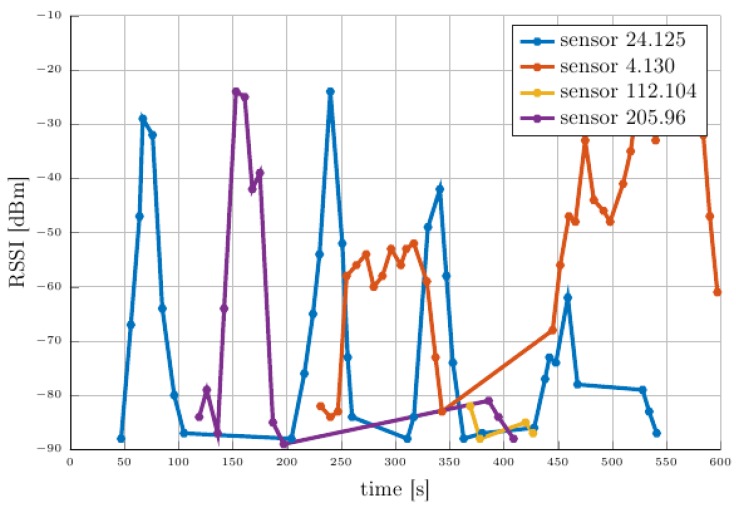
RSSI for each unicast along the experimental run.

Variability of vegetation density during the year, weather conditions, and antenna orientation after uncontrolled aerial deployment are known as critical factors impacting node communication performance [15,16]. In our scenario through the experimental deployment we observe such phenomena for sensor node 4.130, graphically illustrated in Figure 16, which has been deployed at the edge of the field with surrounding vegetation. This particular sensor node also illustrates the induced variability through vegetation effects in the wireless communication performance. To mitigate such situations a probabilistic approach can be implemented which establishes reliable bounds on the RSSI values and availability of the sensor nodes. Also, areas with reduced RSSI can be compensated by increasing the node coverage density e.g., increasing the number of nodes in a given area.

Using the data available from the UAV’s node logs we further analyze the RSSI evolutions illustrated in Figure 16. It can be seen how the RSSI’s evolution changes depending on the position of the UAV.

## 4. Discussion

In this paper we proposed a hybrid UAV-WSN system, which, compared with [19], improves the monitoring of large areas and is dedicated especially to agricultural applications. Monitoring of large geographical areas require efficient data collection strategies, based on collaborative entities, able to overcome the challenges faced and achieve an improved overall system. The main challenges introduced by sparse WSN deployment, which creates an uneven distribution of sensing nodes, were pointed out. Starting from the hypothesis of an unknown sensor distribution over the area of interest, we have defined a strategy that starts with a photogrammetry procedure in which the surface of interest is crossed repeatedly by an UAV to estimate the sensors’ position and receive information required for cluster grouping. Once this data becomes available, a scoring system can be called through which most suitable sensor nodes are proposed for clusters’ head positions. The next step consists of running the clustering algorithm within the WSN nodes. Then we plan a trajectory which passes through the neighbourhoods of the cluster heads with guaranteed communication time and guaranteed obstacle avoidance.

As stated earlier, the main interest of this paper lies in the interplay between various components of the overall heterogeneous WSN surveillance. In particular, we are interested in how the trajectory generation procedures combine with the WSN communication restrictions. Beyond the directions studied in this paper we may consider as being worthwhile for further study:

- The data acquired from the initial, sensor localization, trajectory may be used in the configuration of the sensor clusters (to account for geometrical restrictions: line of sight; distance between nodes, etc.).
- Dynamic reconfiguration of the data-gathering trajectory: each cluster provides a list of potential cluster heads and multiple trajectories are computed (for various combinations of cluster heads).
- Ultimately, we may consider a WSN in which the ground network and the UAV collaborate dynamically to create clusters and design the data-gathering trajectory (with the result depending and being updated by the current UAV position, sensor battery charge and amount of data to be transferred within the network).

All of the above require advanced optimization tools in order to handle the (usually conflicting) constraints. This raises interesting questions of memory and power resources; feature extraction and communication restrictions (capacity, safety and the like).

## 5. Conclusions

Throughout the paper we have shown that trajectories which pass sufficiently near the estimated positions of the cluster heads and, simultaneously avoid the obstacles, can be computed via a mixed-integer based optimization procedure. The proposed clustering mechanism enables effective WSN topology subsystems, optimized for energy consumption balance and fast data exchange. Our experimental results obtained both from comparative simulations and from real implementation showed that this collaborative approach for wide area monitoring is feasible and effective.

## Figures and Tables

**Table 1:** Statistic simulation results.

WSN Size (No. of Nodes)	Power (mW)			Time (s)		
	Max Probability	Mean Value	Standard Deviation	Max Probability	Mean Value	Standard Deviation
40	18	31.5	12.2	30	30.7	22.7
80	55	61.6	12.7	25	24.1	16.3
100	52	57.8	13.1	20	21.9	13.5

**Table 2:** CM3300 main specifications.

Processor	TI MSP430F1611, 48 kB program flash, 10 kB data RAM, 1 MB external flash
RF chip	TI CC2420, IEEE 802.15.4 compliant 2.4~2.485 GHz
Sensitivity	−95 dBm typ
Transfer rate	250 Kbps
RF power	−25 dBm~20 dBm configurable
Range	~800 m (outdoor), ~100 m (indoor) with 5 dBi antenna
Power	3 V–2 AA batteries

**Table 3:** DJI Matrice 600 PRO UAV main specifications.

Dimensions	1608 × 1518 × 727 mm^3^
Weight	9.5 kg
MTOW	15.5 kg
Max speed	65 km/h
Autonomy	32 min
Operating range	5 km
Service ceiling	2500 m
